# Supplementary material for: The interactome of intact mitochondria by cross-linking mass spectrometry provides evidence for coexisting respiratory supercomplexes
Source: Mol Cell Proteomics. 2017 Dec 8;17(2):216–32. doi: 10.1074/mcp.RA117.000470 (PMC5795388; doi:10.1074/mcp.RA117.000470)
Supplement: Supplemental Data [file supp_RA117.000470_133922_0_supp_23978_fzffwf.zip › spectra_annotation/mito_DR_spectra_annotation/6-1-45-1-8-1.pdf]

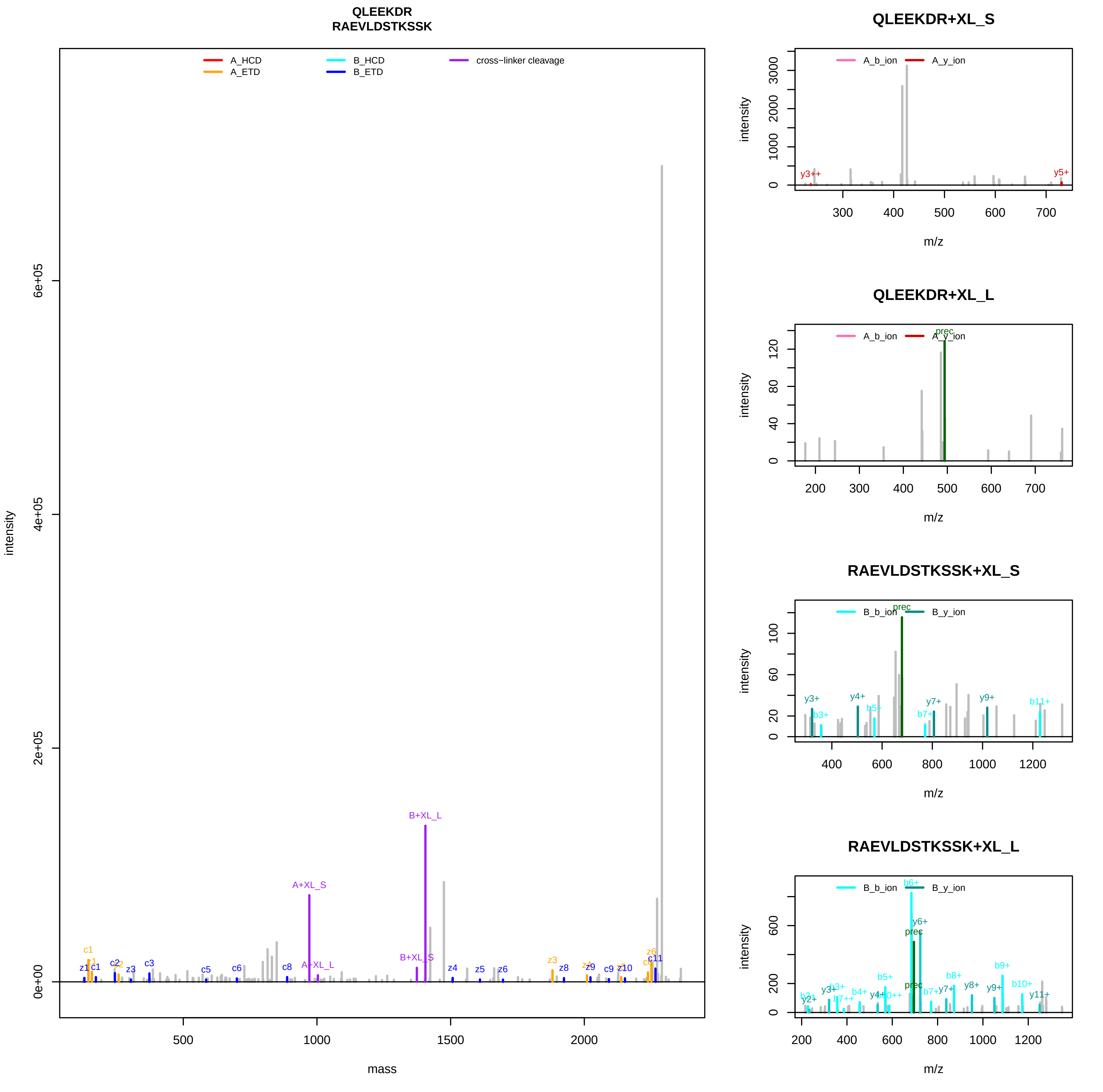
QLEEKDR
RAEVLDSTKSSK
A_HCD
A_ETD
B_HCD
B_ETD
cross−linker cleavage
intensity
mass
B+XL_L
A+XL_S
A+XL_L
B+XL_S
0e+00
2e+05
4e+05
6e+05
500
1000
1500
2000
QLEEKDR+XL_S
A_b_ion
A_y_ion
y3++
y5+
m/z
QLEEKDR+XL_L
prec
RAEVLDSTKSSK+XL_S
B_b_ion
B_y_ion
y3+
b3+
y4+
b5+
b7+
y7+
y9+
b11+
RAEVLDSTKSSK+XL_L
b6+
y6+
b9+
b8+
b10+
y8+
y11+
